# Cardiac herniation six years after thoracoscopic thymectomy: a case report

**DOI:** 10.1186/s40981-026-00856-6

**Published:** 2026-03-24

**Authors:** Yusei Ishizuka, Taiichiro Hayashida, Tomoaki Yoshii, Maiko Hasegawa-Moriyama

**Affiliations:** 1 Department of Anesthesiology, Tsuruoka Municipal Shonai Hospital, 4-20 Izumi-Machi, Tsuruoka, Yamagata 997-8515 Japan; 2 Department of Anesthesiology, Chiba University Hospital, 1-8-1 Inohana-Cho, Chuo-Ku, Chiba, 260-8670 Japan; 3 Department of Anesthesiology, Teikyo University Chiba Medical Center, 3426-3 Anegasaki, Ichihara, Chiba 299-0111 Japan

**Keywords:** Cardiac herniation, Herniation of the heart, Thoracoscopic thymectomy, Myasthenia gravis, Coronary artery bypass grafting

## Abstract

**Background:**

Cardiac herniation is a rare but potentially fatal complication that may occur after surgery involving pericardial incision or resection. Most cases develop intraoperatively or in the early postoperative period, and delayed-onset cases after minimally invasive thoracic surgery have not been reported.

**Case presentation:**

A 48-year-old woman developed left shoulder pain and nausea six years after thoracoscopic thymectomy for myasthenia gravis. Electrocardiography indicated ST-segment elevation myocardial infarction. Computed tomography of the chest showed cardiac herniation into the left thoracic cavity and coronary angiography revealed stenosis corresponding to the hernial defect. The patient was diagnosed with coronary artery compression due to cardiac herniation and underwent hernia reduction and coronary artery bypass grafting. The postoperative course was uneventful.

**Conclusions:**

Cardiac herniation may occur even long after minimally invasive thoracic surgery and should be considered in patients presenting with chest symptoms or hemodynamic instability.

## Background

Cardiac herniation is a serious complication that may occur following any pericardial injury, including pericardial resection or incision. This pathology was first reported in 1948 as a postoperative complication after lung resection with pericardiectomy [1]. Subsequently, cardiac herniation has been described as a rare consequence of thoracic trauma [2] or pneumonectomy [3]. More recently, cardiac herniation has been recognized as a complication of minimally invasive thoracic surgery, such as robot-assisted thymectomy [4] and minimally invasive cardiac surgery (MICS) [5–8]. Most reported cases occur in the acute postoperative period, with the majority developing within 24–48 h after surgery [9]. In contrast, reports of delayed cardiac herniation are exceedingly uncommon [10–14], and onset six years postoperatively, as observed in the present case, is exceptionally rare. Importantly, delayed cardiac herniation following minimally invasive thoracic surgery has not been reported previously.

## Case presentation

A 48-year-old woman (height, 156 cm; weight, 67.6 kg) with a history of dyslipidemia underwent video-assisted thoracoscopic extended thymectomy for myasthenia gravis. The procedure was performed via a bilateral thoracoscopic approach, and no intraoperative pericardial injury was reported.

Six years postoperatively, she presented with left shoulder pain and nausea. Electrocardiography at a referring hospital revealed ST-segment elevation in leads I, II, aVL, and V1–V4, raising suspicion of ST-elevation myocardial infarction. However, computed tomography (CT) demonstrated cardiac herniation into the left thoracic cavity. Coronary angiography revealed significant coronary artery stenosis corresponding to the hernia orifice, leading to the diagnosis of coronary artery compression secondary to cardiac herniation. Given the hemodynamic stability of the patient, the resolution of symptoms, and the potential need for surgical intervention, percutaneous coronary intervention was deferred. Instead, she was admitted for careful hemodynamic monitoring, and pharmacotherapy was initiated with an oral coronary vasodilator. She was subsequently referred to our institution for further evaluation, including surgical indications.

**Fig. 1:**
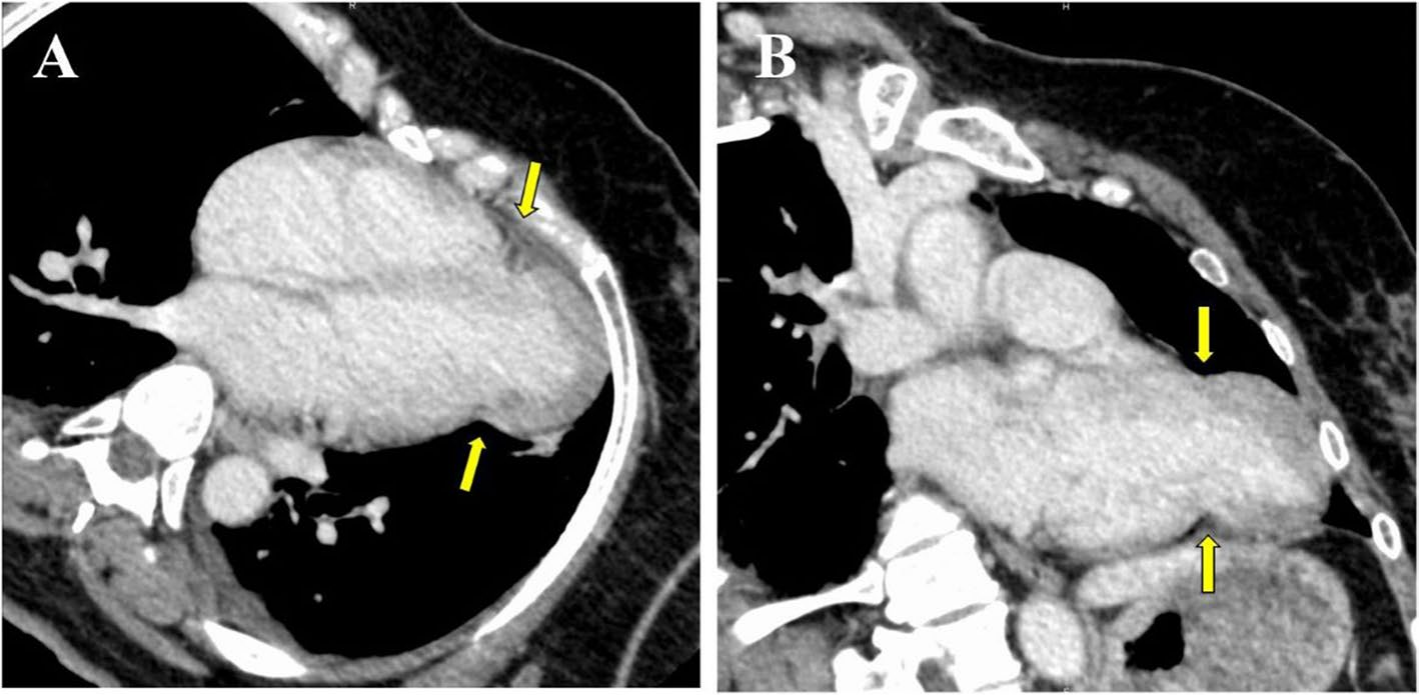
Contrast-enhanced computed tomography of the chest. Axial (**A**) and sagittal (**B**) views showing cardiac herniation into the left thoracic cavity from the mid-left ventricle to the apex (yellow arrow)

Chest radiography at our hospital showed no apparent abnormalities. However, contrast-enhanced CT revealed herniation of the left ventricle from the mid-ventricular to apical region into the left thoracic cavity (Fig. 1).

**Fig. 2:**
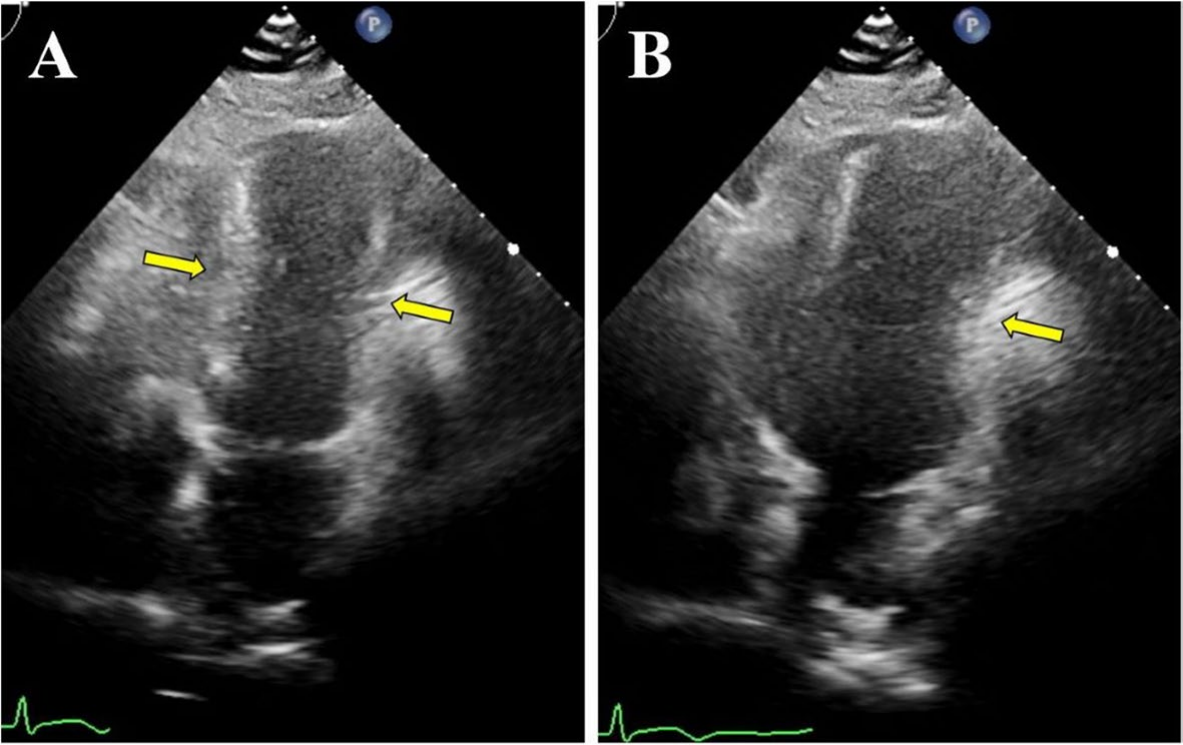
Transthoracic echocardiography. Apical four-chamber views in systole (**A**) and diastole (**B**) show both ventricles are compressed from the outer side (yellow arrow)

Transthoracic echocardiography demonstrated external compression of both ventricles and mild hypokinesis from the mid-ventricular to the apical segments (Fig. 2).

**Fig. 3:**
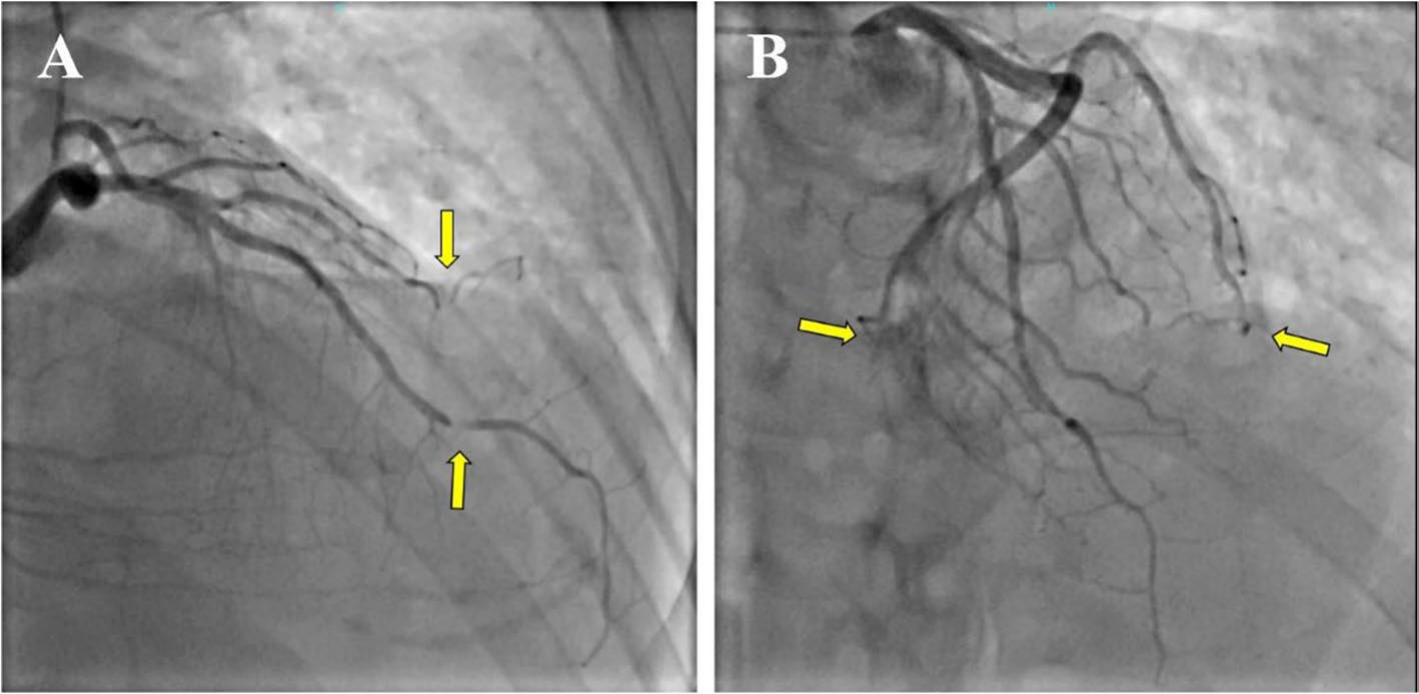
Coronary angiography. Coronary artery stenosis is observed through the hernial orifice (yellow arrow)

Coronary angiography showed that the posterolateral branch was occluded, while the distal segment of the left anterior descending artery (LAD), 2nd diagonal branch, and obtuse marginal branch had stenosis of approximately 99%, all corresponding to the hernia orifice (Fig. 3).

**Fig. 4:**
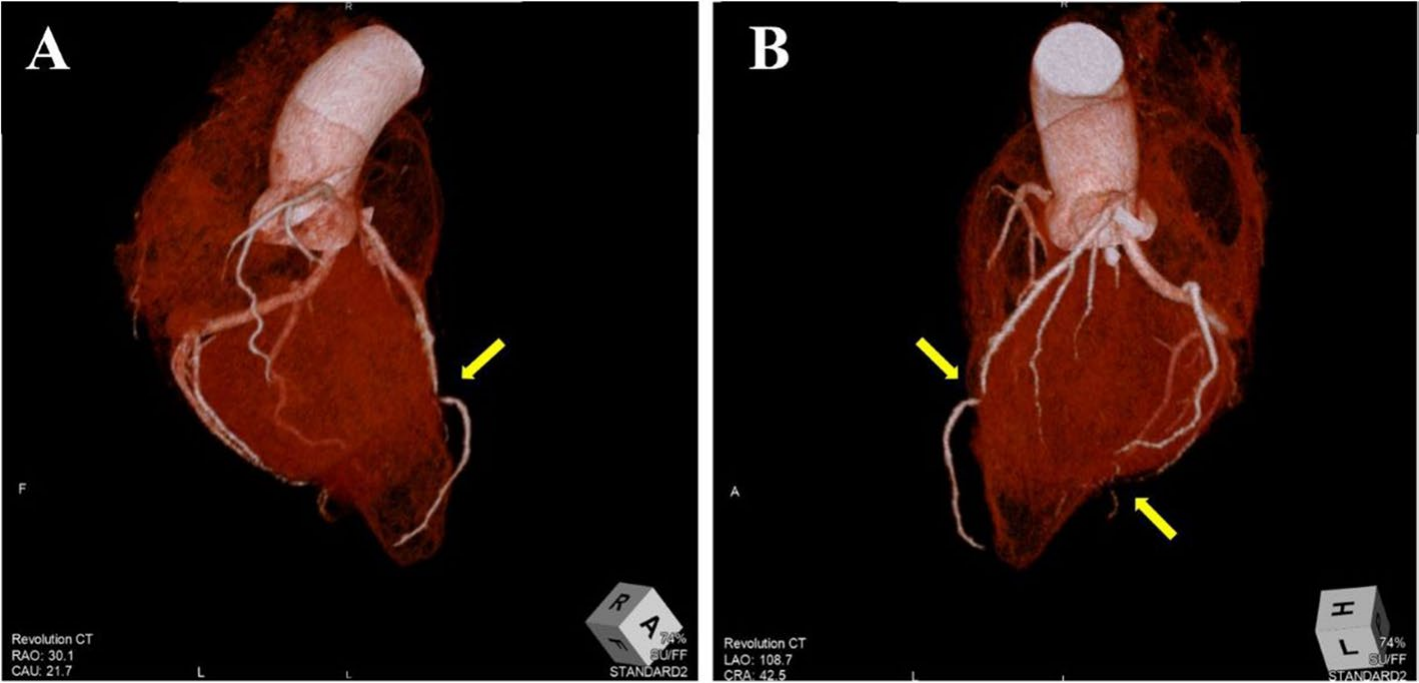
Computed tomography of the heart. Stenosis is observed at same sites identified on coronary angiography

Cardiac CT angiography confirmed these findings (Fig. 4).

**Fig. 5:**
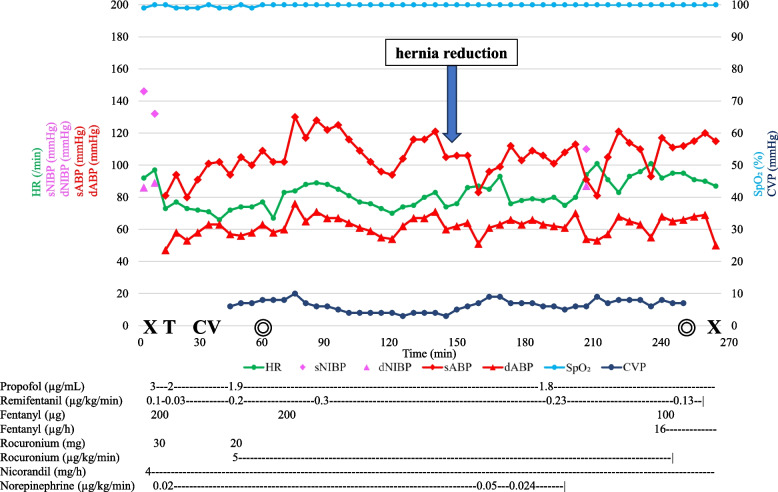
Anesthesia record. As there were minimal changes in hemodynamic parameters during hernia reduction, norepinephrine was maintained at 0.02 µg/kg/min until the initiation of CABG without requiring other vasoactive agents. During subsequent cardiac retraction, the norepinephrine infusion rate was temporarily increased, however the infusion was successfully discontinued before the completion of surgery. HR, heart rate; sNIBP, systolic noninvasive blood pressure; dNIBP, diastolic noninvasive blood pressure; sABP, systolic arterial blood pressure; dABP, diastolic arterial blood pressure; SpO₂, percutaneous oxygen saturation; CVP, central venous pressure; X, start or end of anesthesia; T, intubation; CV, central venous catheter insertion; ◎, start or end of surgery

As chest symptoms improved with conservative management and hemodynamic status remained stable, the patient underwent elective surgical repair of the cardiac hernia and coronary artery bypass grafting (CABG) two months after symptom onset. The time course of hemodynamic parameters and pharmacological interventions during general anesthesia is illustrated in Fig. 5.

**Fig. 6:**
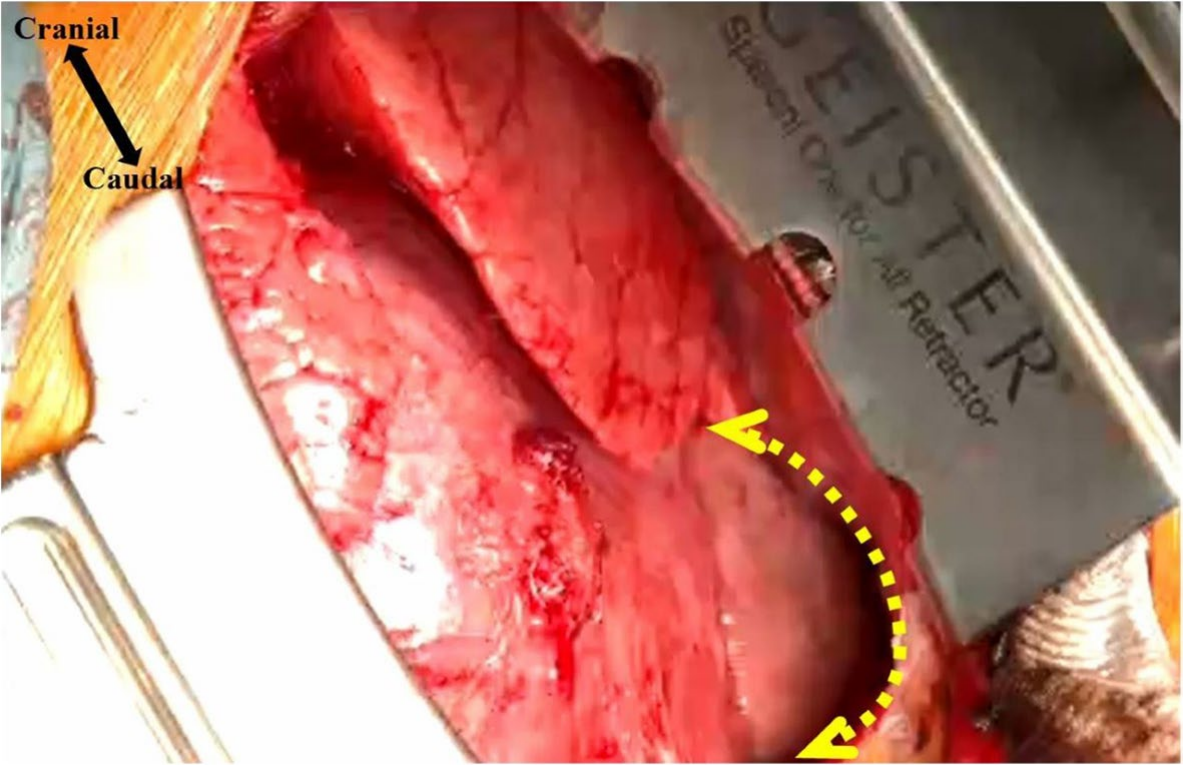
Surgical findings. The heart is seen protruding from the pericardial defect (yellow arrow)

From the induction of anesthesia, nicorandil 4 mg/h was administered via continuous intravenous infusion, together with norepinephrine 0.02 µg/kg/min to maintain hemodynamic stability. After preoxygenation, general anesthesia was induced with propofol at a target plasma concentration of 3 μg/mL using a Terumo® infusion pump equipped with Diprifusor software implementing the Marsh model, fentanyl 200 μg, remifentanil 0.1 μg/kg/min, and rocuronium 30 mg. Following endotracheal intubation with an ID 7.0 mm endotracheal tube, an arterial pressure line was established via the right radial artery. After placement of a transesophageal echocardiography (TEE) probe, an oximetric central venous catheter was inserted via the right internal jugular vein for central venous pressure monitoring and medication administration. Anesthesia was maintained with propofol 1.8–3.0 µg/mL, remifentanil 0.03–0.3 µg/kg/min, and a total of 500 µg of fentanyl. Norepinephrine 0.02–0.05 μg/kg/min was administered to maintain blood pressure. Intraoperative findings confirmed cardiac herniation through a pericardial defect into the left thoracic cavity (Fig. 6).

Following hernia reduction, off-pump CABG was performed using the left internal thoracic artery to the LAD. To confirm cardiac compression at the hernia orifice, the heart was temporarily retracted, during which the norepinephrine infusion rate was increased from 0.02 to 0.05 µg/kg/min (Fig. 5). The infusion was discontinued before the completion of surgery. The pericardial defect was repaired with a Gore-Tex® patch. During hernia reduction, rebound hypertension and bradycardia were anticipated and antihypertensive agents were prepared. However, as hemodynamic parameters remained stable throughout hernia reduction, these vasoactive agents were not required. Continuous TEE monitoring was performed, with no significant changes observed. She was admitted to the intensive care unit and extubated on postoperative day 0. The postoperative course was uneventful, and she was discharged on postoperative day 7 without complications.

## Discussion

Cardiac herniation may result from congenital, traumatic, or iatrogenic causes, and the clinical manifestations range widely from no symptoms subjects to sudden cardiac death [15]. The pathophysiology varies by case, depending on the presence and combination of cardiac displacement, torsion of the great vessels, associated inflow or outflow obstructions, and strangulation at the herniation orifice [10]. The hemodynamic consequences are influenced by the location and size of the herniation defect. Right-sided cardiac herniation is more likely to cause reduced venous return and cardiac output due to torsion at the atrioventricular junction and bilateral ventricular outflow tracts [15]. In contrast, left-sided cardiac herniation tends to result in myocardial and coronary artery compression at the herniation orifice [15]. In the present case, which involved left-sided cardiac herniation, acute myocardial infarction was presumed to have occurred due to coronary artery stenosis caused by compression at the herniation site.

Cardiac herniation is classified into complete and partial types. Partial herniation is considered to carry a poorer prognosis than complete herniation, given the higher risk of cardiac incarceration and myocardial ischemia [10]. However, the present case of partial herniation demonstrated a favorable clinical course. This outcome may be attributed to the fact that the herniation resulted in only limited stenosis of the coronary artery without significant circulatory instability, and that the myocardial ischemia caused by coronary compression was reversible following coronary artery bypass and decompression.

Reports describing anesthetic management in patients with cardiac herniation are extremely rare. We therefore anticipated potential circulatory changes associated with herniation reduction. Before reduction, cardiac function was considered mechanically restricted. Therefore, to prevent circulatory collapse due to anesthesia-induced afterload reduction, continuous administration of vasoconstrictors was planned from the induction of anesthesia to maintain systemic vascular resistance. At the time of hernia reduction, we anticipated that the release of cardiac restriction could lead to increased preload and an abrupt elevation of blood pressure. Surgical relief of cardiac tamponade, which similarly involves the release of cardiac restriction, is known to provoke rebound hypertension and bradycardia [16]. Although we expected comparable circulatory fluctuations during herniation reduction, no significant hemodynamic changes were observed. This suggests that in cases with minimal preoperative circulatory instability, the physiological changes accompanying herniation reduction may also be limited.

Cardiac herniation typically presents within 24–48 h postoperatively [9]. However, delayed cases diagnosed months to years after surgery have also been reported [12, 14]. Although a case of progressive dyspnea lasting over a year has been reported [14], that patient had experienced only several brief episodes of chest pain up to the point of seeking medical attention, and few findings suggested progression of the cardiac hernia. No cases of chronic-phase cardiac herniation have been documented following thoracoscopic surgery or MICS. The present case, in which the patient presented six years after thoracoscopic thymectomy, highlights the need to recognize cardiac herniation as a potential late complication of such procedures. The precise mechanisms underlying such delayed onset remain unclear. Given the limited operative field and visualization inherent to thoracoscopic surgery, a small, unrecognized pericardial defect may plausibly have gradually enlarged over time, ultimately resulting in cardiac herniation. Meticulous intraoperative inspection of the pericardium and prophylactic closure or reinforcement of even minor defects, when identified, may represent potential preventive measures.

In recent years, the number of minimally invasive procedures, including robot-assisted and thoracoscopic surgeries, has been increasing due to the clear advantages in reducing postoperative pain and promoting early recovery. This case represents an exceptionally rare instance of cardiac herniation manifesting six years after thoracoscopic thymectomy and serves as an important reminder of the potential risk of delayed cardiac herniation in the chronic postoperative phase following minimally invasive thoracic surgery.

## Data Availability

Data sharing is not applicable to this article as no datasets were generated or analyzed during the current study.

## Abbreviations

MICS: Minimally invasive cardiac surgery
CT: Computed tomography
LAD: Left anterior descending artery
CABG: Coronary artery bypass grafting
TEE: Transesophageal echocardiography
